# Impact of a Single Session of Hybrid Carbon Dioxide 10600 Nanometer and 1540 Nanometer Laser With Platelet-Rich Plasma Treatment in the Genital Area to Treat Female Sexual Dysfunction: A Pilot Study

**DOI:** 10.7759/cureus.75990

**Published:** 2024-12-19

**Authors:** Jose M Togo, Thania M Hurtado

**Affiliations:** 1 Department of Laser, Regenerative, and Functional Gynecology, Central Medica Quirurgica, Mazatlan, MEX; 2 Department of Gynecologic Oncology, Central Médica Quirúrgica, Mazatlan, MEX; 3 Department of Aesthetic and Anti-Aging Medicine, Central Medica Quirurgica, Mazatlan, MEX

**Keywords:** dyspareunia, female sexual dysfunction, fractional microablative co2 vaginal laser, genitourinary syndrome of menopause, hybrid laser therapy, platelet-rich plasma

## Abstract

Background

Female sexual dysfunction (FSD) affects a significant portion of the female population, negatively impacting quality of life. New therapeutic approaches, such as the combination of laser therapy and platelet-rich plasma (PRP), are being explored as potential treatments to enhance sexual function in affected women.

Methods

This original study involved 23 women aged 37 to 72, all diagnosed with varying degrees of FSD (mild, moderate, severe). The participants underwent a pre-treatment evaluation using a custom-designed Likert scale (Female Sexual Health Questionnaire) designed by the authors to evaluate the symptoms of the patients. The treatment involved a single session combining hybrid carbon dioxide 10600 nm and 1540 nm laser therapy with PRP applied to the genital area. One month post-treatment, participants were reassessed using the same questionnaire.

Results

Of the 23 patients studied, 65.2% (15 patients) exhibited hypoactive sexual desire, and 73.33% of them (11 out of 15) demonstrated improvement post-treatment (P = 0.00048). Overall sexual satisfaction increased by 28.2% (P = 0.0032), and lubrication improved in 54.5% of patients (P = 0.0027). Significant reductions in dyspareunia were observed, with 80% of patients experiencing pain relief (P = 0.0035). Patients with moderate dysfunction showed the highest response, with 100% (6 out of 6) demonstrating improvement across domains (P = 0.0009). Postmenopausal women experienced a 30.9% improvement in sexual function (P = 0.007), while premenopausal women exhibited a 7.5% improvement (P = 0.04). Statistically significant changes were also noted in the frequency of orgasm (P = 0.0058) and genital sensitivity in patients with moderate dysfunction.

Conclusion

Our data indicated significant improvements in FSD, particularly in moderate to severe dysfunction, with positive changes in key aspects of sexual function such as lubrication, desire, and ease of orgasm. However, the limited number of participants and short follow-up in this pilot study highlight the need for further investigation into hybrid CO2/1540 nm laser therapy combined with PRP as a therapeutic option for female sexual dysfunction.

## Introduction

Female sexual dysfunction (FSD) encompasses a series of problems that affect desire, arousal, orgasm, and pain during sexual intercourse. These issues are often persistent or recurrent, leading to significant emotional distress and relationship difficulties [1]. FSD can occur at any stage of life, with a considerable impact on women's quality of life [1-3]. Specific disorders include sexual interest/arousal disorder, female orgasmic disorder, genito-pelvic pain/penetration disorder, and drug-induced sexual dysfunction [2]. The prevalence of these disorders is high, affecting a large proportion of women worldwide [4].

The diagnosis of FSD requires a comprehensive evaluation, including medical and sexual history, and, in some cases, a physical examination [4]. A crucial factor in defining a sexual issue as dysfunction is the personal distress it causes [5]. In postmenopausal women, genitourinary syndrome of menopause (GSM), characterized by symptoms such as vaginal dryness, irritation, dyspareunia, and urinary incontinence, is a common contributor to FSD [5].

Carbon dioxide (CO2) laser treatment has been extensively studied as a therapeutic option for FSD, particularly in cases of GSM and vaginal laxity [6-10]. Studies have documented significant improvements in vaginal health and sexual function, assessed through validated indices such as the Vaginal Health Index (VHI) and the Female Sexual Function Index (FSFI) [7-11]. For instance, CO2 laser treatment has been associated with enhancements in lubrication, sexual desire, and reductions in pain during sexual activity [12-13]. Additionally, in women with vulvovaginal atrophy and breast cancer survivors, CO2 laser therapy has demonstrated its potential to alleviate symptoms like dryness and dyspareunia, improving overall quality of life [14-16].

Despite the documented benefits of CO2 laser therapy, its reliance on multiple sessions to achieve optimal and sustained outcomes poses a practical challenge. This limitation has led to the exploration of hybrid technologies, such as the combination of CO2/1540 nm lasers and Platelet-Rich Plasma (PRP). These approaches aim to enhance therapeutic efficacy while potentially reducing the number of sessions required, providing a more efficient solution for patients with FSD.

This pilot study was designed to evaluate the feasibility and efficacy of a single-session hybrid CO2/1540 nm laser treatment combined with PRP for FSD. By focusing on key parameters such as lubrication, sexual desire, frequency and ease of orgasm, and reduction of pain during sexual activity, the study aims to provide preliminary evidence for the potential advantages of this innovative therapeutic strategy.

## Materials and methods

Study design

This prospective, interventional, and descriptive pilot study was conducted from July 13, 2023, to August 30, 2024, to evaluate the preliminary effectiveness of a single session of hybrid CO2 10600 nm/1540 nm laser (Deka μ-Scan V2LR, Deka M.E.L.A., Calenzano, Italy) combined with platelet-rich plasma (PRP) treatment at the genital level to improve female sexual dysfunction. The severity of sexual dysfunction (mild, moderate, or severe) was determined using a custom-designed Female Sexual Health Questionnaire developed by the authors, which evaluates key dimensions of sexual health (detailed below). The study included patients presenting with symptoms such as reduced lubrication, decreased sexual desire, pain during sexual activity, and difficulty achieving orgasm.

Study population

A total of 23 patients, aged between 37 and 72 years, participated in the study. All had a prior diagnosis of sexual dysfunction at mild, moderate, or severe levels.

Inclusion criteria

The study included adult women aged 18 years and older with diagnosed sexual dysfunction who maintained an active sexual life. Eligible patients had not received genital laser treatments, PRP, or local/systemic hormone replacement therapy within the past six months. All participants completed the Female Sexual Health Questionnaire designed by the team both before the first treatment session and again at a follow-up evaluation conducted four weeks later.

Exclusion criteria

Exclusion criteria included women with medical conditions such as uncontrolled diabetes, autoimmune diseases, active infections, cardiovascular diseases (e.g., heart failure or unstable angina), or cancer under active treatment. Psychiatric conditions included major depressive disorder, bipolar disorder, schizophrenia, or any other disorder that could interfere with evaluating treatment outcomes. Pregnant or breastfeeding women and those not meeting the inclusion criteria were also excluded.

Intervention

The treatment protocol consisted of a single session using the hybrid Deka Duo Glide CO2/1540 nm laser (Deka M.E.L.A., Calenzano, Italy) applied to the vaginal area, combined with PRP injections at specific genital sites. PRP was administered to the anterior vaginal wall (4 cc), the clitoris (1 cc), and the vaginal introitus (2 cc). Laser parameters for MonaLisa Glide (Deka M.E.L.A., Calenzano, Italy) included a wavelength of 10600 nm at 40 watts and 1540 nm at 8 watts, with a Deka pulse, a dwell time of 1000 µs, a dot spacing of 1000 µm, and a smart stack set to 1. A 360-degree handpiece was used, rotating it 15 degrees across three positions, retracting by 1 cm after each pass. For the vaginal introitus, a hybrid beam of 10600 nm at 30 watts plus 1540 nm at 4 watts was applied, with a dwell time of 400 µs, a dot spacing of 1000 µm, and a smart stack set to 2. Subsequently, PRP was injected into the indicated sites.

Assessment of adverse effects

No adverse effects were identified during the study period. Although adverse effects were not formally assessed through a structured tool, patients were clinically monitored throughout the study to detect any potential treatment-related events. Based on previous literature, expected adverse effects included mild vaginal irritation, erythema, or transient discomfort at the injection sites; however, none were reported during follow-up.

Evaluation questionnaire

To assess sexual function variables, a custom-designed Likert scale questionnaire (Female Sexual Health Questionnaire) was developed by the authors, specifically tailored for this study to simplify patient responses while effectively capturing key aspects of female sexual health. While inspired by the general structure of the Female Sexual Function Index (FSFI) [17], this questionnaire is an original creation, designed independently and not a direct modification of the FSFI.

Differences from FSFI

The FSFI evaluates six domains of sexual function through 19 questions. In contrast, our questionnaire consolidates these into seven dimensions assessed through 10 simplified questions. The scoring scale ranges from 2 to 10 points per question to facilitate clarity and accuracy during clinical evaluations.

The validity of the questionnaire was assessed through face and content validity methods. Face validity was ensured by independent reviews from three experts in gynecology, sexual medicine, and psychology, who confirmed that the questions were clear, relevant, and appropriate for the study's objectives. The questionnaire evaluates seven dimensions of sexual health: sexual desire, genital sensitivity, lubrication, orgasm frequency, ease of achieving orgasm, pain during sexual activity, and overall sexual satisfaction.

Each dimension was scored from 2 to 10 points, with higher scores indicating improved sexual health. The questionnaire was administered to patients before and after the treatment, with an average follow-up interval of four weeks. Patients were classified into categories of sexual dysfunction based on the total score: severe (14-34 points), moderate (35-48 points), mild (49-63 points), and no dysfunction (64-70 points).

Statistical analysis

Changes in variables before and after treatment were evaluated using the Student’s t-test for dependent samples, as the data consisted of continuous measurements (pre- and post-treatment scores). Additionally, percentage improvement in each variable was calculated for patients with severe, moderate, and mild sexual dysfunction.

Statistical analyses were performed using Microsoft Excel for basic calculations and Python (version 1.10.7; Python Software Foundation, Delaware, USA) on Python Code Pad for advanced statistical analyses. The Student’s t-test for dependent samples was used to compare pre- and post-treatment variables, with results considered statistically significant at P < 0.05.

## Results

The 23 studied patients were classified according to their degree of sexual dysfunction based on the Female Sexual Health Questionnaire as follows (Table 1): eight patients (34.8%) with severe sexual dysfunction (mean score of 28.25), eight patients (34.8%) with moderate sexual dysfunction (mean score of 43), and seven patients (30.4%) with mild sexual dysfunction (mean score of 54.57).

**Table 1:** Distribution of Patients Based on the Severity of Sexual Dysfunction Grade of Sexual Dysfunction

Grade of Sexual Dysfunction	Number of Patients (%) Total Patients=23
Mild (49-63 points)	7 (30.4%)
Moderate (35-48 points)	8 (34.8%)
Severe (14-34 points)	8 (34.8%)

The included patients aged between 37 and 72 years, with an average age of 51 years. The majority of participants (95.7%) were Hispanic, and one patient (4.3%) was White. All participants reported an active sexual life. Regarding hormonal status, 15 patients (65.2%) were postmenopausal, and eight patients (34.8%) were premenopausal.

Regarding reproductive history, 21 patients (91.3%) reported having had pregnancies, with an average of three pregnancies. Additionally, 13 patients (56.5%) had given birth, with an average of 2.38 births among those who had experienced childbirth. Six patients (26.1%) had undergone cesarean sections, with an average of 1.75 cesarean sections among these patients. Five patients (21.7%) reported having experienced miscarriages, with an average of 1.56 miscarriages among them. In terms of surgical history, four patients (17.4%) had a history of hysterectomy, and four patients (17.4%) had previous vaginal surgeries, including colpoperineoplasty or suburethral mesh placement.

The treatment with hybrid CO2/1540 nm laser and PRP showed a significant improvement in overall sexual dysfunction in patients across different initial severity levels (Table 2).

**Table 2:** Improvement of dysfunction degree Note: P-values were calculated using the Student’s t-test for paired samples.

Change of Dysfunction Degree	Number of Patients that Improved (%)	P-value
Severe to Moderate/Mild	5 (62.5%)	0.0077
Moderate to Mild	6 (75%)	0.026

The improvement rate was also calculated according to the patient's hormonal status (pre/postmenopausal), showing an improvement in both groups, especially in postmenopausal women, with a 30.9% improvement in the questionnaire score (p: 0.007) and a 7.5% improvement in premenopausal women (p: 0.04) (Table 3).

**Table 3:** Hormonal Status of the Patients Note: P-values were calculated using the Student’s t-test for paired samples

Hormonal Status	Pre-Treatment Average Score	Post-Treatment Average Score	Difference/Improvement (%)	P-value
Premenopausal	46.92	50.46	7.50%	0.04
Postmenopausal	34.2	44.8	30.90%	0.007

It is worth noting that among premenopausal patients, five (38.5%) had mild sexual dysfunction, five (38.5%) had moderate dysfunction, and three patients (23.1%) had severe dysfunction. A total of eight out of 13 patients (61.5%) showed improvement in their scores. This contrasts with the seemingly minimal average improvement percentage observed in this group, which can be explained by the fact that premenopausal patients had higher initial scores compared to postmenopausal patients (46.9 points vs. 34.2 points, respectively).

Lubrication

Out of the 23 patients studied, 22 (95.7%) reported a lubrication score of 8 or less. Among these, four patients (18.2%) presented a score of 2, indicating severe dryness; eight patients (36.4%) reported minimal lubrication with a score of 4; six patients (27.3%) described partial lubrication during sexual activity, with a score of 6; and four patients (18.2%) indicated being lubricated throughout sexual activity, with a score of 8. The average pre-treatment score among these 22 patients was 4.91 (Table 4).

**Table 4:** Improvement of Lubrication (Overall) Note: P-values were calculated using the Student’s t-test for paired samples.

Degree of Sexual Dysfunction (Number of patients with pain)	Initial Score (lubrication)	Score after Treatment (lubrication)	Number of patients that improved (%)	P-value
All Degrees of Sexual Dysfunction (22)	4.91	6.45	12 (54.5)	0.0027

Post-treatment, 12 of the 22 patients (54.5%) showed an improvement in their lubrication scores (P = 0.0027). Among these 12 patients, six (50%) reported being well-lubricated or very lubricated after the intervention. Five patients (41.7%) improved their scores to 6 points, indicating partial lubrication during the sexual act, and one patient moved from being very dry to slightly lubricated. Of the 10 patients (45.5%) who did not show changes in their scores, three (30%) had an initial score of 8 points (well-lubricated), three (30%) reported partial lubrication (4 points), and four (40%) had an initial score of 6 points. The average pre-treatment score among these 22 patients was 4.91, which increased significantly after the treatment, demonstrating the effectiveness of the intervention in enhancing lubrication (Table 5).

**Table 5:** Improvement in Lubrication Based on Initial Scores This table illustrates the percentage of patients who showed improvement in lubrication after treatment, categorized by their initial lubrication scores. Lubrication scores were assessed as follows: 2 points: Very dry (severe lack of lubrication throughout sexual activity). 4 points: Poorly lubricated (minimal lubrication, requiring additional efforts or discomfort during sexual activity). 6 points: Partially lubricated (adequate lubrication at some points during sexual activity but not consistently throughout). 8 points: Well lubricated (sufficient lubrication for comfortable sexual activity). 10 points: Very well lubricated (abundant lubrication throughout sexual activity).

Initial Score	Number of Patients	Improved Patients	Improvement Percentage (%)
2	3	3	100%
4	7	4	57.14%
6	7	3	42.86%
8	5	2	40%

Sexual desire

Of the 23 patients studied, 15 (65.2%) exhibited hypoactive sexual desire, defined as a score below 6 (average initial score of 4.1). This condition was universally present in the group with severe sexual dysfunction, where all eight patients (100%) reported low sexual desire (basal score of 3.5). In the moderate dysfunction group, five out of eight patients (62.5%) demonstrated hypoactive sexual desire (initial score of 4.8). Similarly, two out of seven patients (28.57%) in the mild dysfunction group also exhibited this issue (initial score of 5) (Table 6).

**Table 6:** Effect of Hybrid CO₂ 10600 nm/1540 nm Laser Combined with Platelet-Rich Plasma (PRP) on Sexual Desire in Patients with Female Sexual Dysfunction Baseline Score (Mean): The mean score of sexual desire prior to treatment, as assessed by a standardized questionnaire. Post-Treatment Score (Mean): The mean score of sexual desire one month following the intervention. Number of Patients Improved (%): The number and percentage of patients in each group who showed improvement in their sexual desire scores. P-Value: Statistical significance of the change in scores, as determined by a paired t-test. A P-value < 0.05 indicates a statistically significant improvement.

Degree of Sexual Dysfunction	Baseline Score (Mean)	Post-Treatment Score (Mean)	Number of Patients Improved (%)	P-Value
All Degrees of Sexual Dysfunction (15)	4.1	6	11/15 (73.33%)	0.00048
Grade of Dysfunction				
Mild (2)	5	6	0/2 (0%)	>0.05
Moderate (5)	4.8	7.2	5/5 (100%)	0.0039
Severe (8)	3.5	5.25	5/8 (62.5%)	0.041

After the intervention, 73.33% (11 out of 15) of patients with hypoactive sexual desire showed improvement, with their scores increasing from an initial average of 4.1 to 6 (P: 0.00048).

In the severe dysfunction group, 62.5% (5 out of 8) of the affected patients showed improvement, with their scores increasing from an initial average of 3.5 to 5.2 (P: 0.041). In the moderate dysfunction group, all five affected patients demonstrated improvement, with 60% (3 out of 5) achieving a score of 8 ("High sexual desire"). The average score for these patients increased from 4.8 to 7.2 (P: 0.0039). In the group of patients with mild sexual dysfunction, of the two individuals who presented with a baseline score of 6 or lower, the patient reporting low sexual desire (4 points) showed improvement, reaching a score of 6 (occasional desire). However, given the small sample size and minimal overall changes, no statistically significant differences were observed in this group.

Genital sensitivity

After one treatment session, patients reported an improvement in this area, with the average score increasing from 5.2 to 6.0, representing an average improvement of 26.4% in the group of studied patients (T-statistic: -2.472; P-value: 0.021) (Table 7).

**Table 7:** Improvement in Genital Sensitivity Based on the Degree of Sexual Dysfunction Note: Genital sensitivity was evaluated on a scale ranging from 2 to 10 points. Note: P-values were calculated using the Student’s t-test for paired samples.

Degree of Sexual Dysfunction (Number of Patients)	Initial Score (Genital Sensitivity)	Score after Treatment (Genital Sensitivity)	Percentage of Improvement (Genital Sensitivity)	P-value
All Degrees of Sexual Dysfunction (23)	5.2	6	26.40%	0.02
Mild (7)	7.14	7.71	8.30%	0.35
Moderate (8)	5.25	6	18.75%	0.19
Severe (8)	3.5	4.75	50%	0.10

All patients with severe sexual dysfunction (8/8) reported low genital sensitivity during sexual activity, with a pre-treatment average score of 3.5 points. Among these, four patients showed improvement in genital sensitivity. The post-treatment average score increased by 50% to 4.75 points; however, this improvement was not statistically significant (P = 0.1).

In the moderate sexual dysfunction group, two out of eight patients showed improvement in this parameter, while the remaining six patients maintained the same score (6 points). The pretreatment score was 5.25, and the score after one month of the intervention was 6, this represented an 18% improvement, although it was not statistically significant (P = 0.19).

In the mild sexual dysfunction group, patients generally exhibited good sensitivity, with an initial average score of 7.1 points. Two patients (28.6%) showed an improvement in their score, five patients (71.4%) maintained the same score of 8 points, and one patient (14.3%) experienced a decrease in sensitivity. These changes were not statistically significant (P = 0.35).

Improvement in pain during sexual activity

Of the 23 patients studied, 15 (65.22%) experienced some degree of dyspareunia (average score of 6.2). Among these, four patients (26.67%) had a score of 4 points (very painful sexual activity), five patients (33.33%) had a score of 6 (painful but tolerable sexual activity), and six patients (40%) had a score of 8 (mildly painful sexual activity).

After treatment, of the 15 patients who had dyspareunia, 12 (80%) reported a reduction in pain during sexual activity with a post-treatment average score of 8.13 (P=0.0035). Five of the 15 patients (33.33%) stated that their sexual activity was now pain-free, while two patients (13.33%) showed no change in their score (Table 8).

**Table 8:** Improvement in Sexual Pain Reduction Based on the Degree of Sexual Dysfunction Note: P-values were calculated using the Student’s t-test for paired samples.

Degree of Sexual Dysfunction (Number of Patients with Pain)	Initial Score (Pain)	Score after Treatment (Pain)	Number of Patients Pain-Free (%)	P-value
All Degrees of Sexual Dysfunction with Pain (15)	6.2	8.13	5 out of 15 (33.33%)	0.0035
Grade of dysfunction (Number of patients)				
Mild (4)	8	9.5	3 out of 4 (75%)	0.05
Moderate (4)	5.5	8	0 out of 4 (0%)	0.07
Severe (7)	5.7	8	2 out of 7 (28.5%)	0.0046

Among the group of patients with severe sexual dysfunction, seven out of eight patients (87.5%) presented with dyspareunia prior to treatment. Of these, two patients (28.6%) had a score of 4 points, indicating very painful sexual activity; four patients (57.1%) reported moderate pain with a score of 6 points; and one patient (14.3%) reported mild pain with a score of 8 points. After treatment, six out of these seven patients (85.7%) showed improvement, while one patient (14.3%) maintained the same score of 6 points. Specifically, three patients (42.9%) progressed to a category of mild pain with a score of 8 points, and another two patients (28.5%) reported pain-free sexual activity with a score of 10 points. The average pre-treatment score for dyspareunia in this group was 5.7 points, which increased to 8 points post-treatment. This improvement was statistically significant, with a calculated P-value of 0.0046, demonstrating the efficacy of the intervention in alleviating pain during sexual activity in this subgroup.

In the group with moderate sexual dysfunction, four out of eight patients (50%) reported some degree of dyspareunia. Of these four patients, two (50%) reported very painful sexual activity with a score of 4 points, one patient (25%) described tolerable pain with a score of 6 points and one (25%) experienced mild pain with a score of 8 points.

After the treatment, the patients who did not report pain before the intervention remained free of dyspareunia. Among the four patients who reported dyspareunia, three (75%) improved, transitioning to a score of 8 points (mild pain), while one patient (25%) maintained the same score of 8 points. These changes were not statistically significant, with a P-value of 0.07.

The group identified with mild sexual dysfunction had the highest initial pre-treatment score, with an average of 8.85 points. Four out of seven patients (57.14%) reported mild pain during sexual activity (8 points), while three patients (42.86%) reported no pain.

After treatment, six out of seven patients (85.71%) reported no dyspareunia, of the four patients that reported dyspareunia, three (75%) reported no pain during sexual activity (P=0.05), while the remaining patient maintained their initial score of 8 points (mild pain). The average post-treatment score was 9.7 points compared to 8.85 points pre-treatment.

Ease of reaching orgasm

Of the 23 patients studied, 16 (69.6%) reported difficulty achieving orgasm. Among these, three patients (18.8%) were unable to reach orgasm (2 points), six patients (37.5%) found it very difficult to reach orgasm (4 points), and seven patients (43.8%) were able to reach orgasm but with significant difficulty (6 points). In contrast, seven patients (30.4%) had no difficulty achieving orgasm, with one patient (4.3%) reporting it was very easy (10 points) and six patients (26.1%) describing it as easy.

After treatment, nine out of the 16 patients (56.3%) showed improvement (P=0.0039). Among these, three patients (18.8%) reported they could now easily achieve orgasm (8 points), five patients (31.3%) indicated they could achieve orgasm but with difficulty (6 points), and one patient (6.3%) who was previously unable to achieve orgasm began experiencing orgasms, albeit with difficulty. Meanwhile, seven patients (43.8%) showed no changes in their ability to achieve orgasm (Table 9).

**Table 9:** Improvement in Ease of Reaching Orgasm Based on the Degree of Sexual Dysfunction Note: Ease of reaching orgasm was evaluated on a scale ranging from 2 to 10 points. Note: P-values were calculated using the Student’s t-test for paired samples.

Degree of Sexual Dysfunction (Number of Patients)	Initial Score	Score After Treatment	Number of Patients that Improved (%)	P-Value
All degrees of sexual dysfunction (16)	5.4	6.2	9 (56.3%)	0.0039
Grade of dysfunction (Number of patients)				
Mild (1)	6	6	0.00%	> 0.05
Moderate (5)	6.5	8	5 (100%)	0.04
Severe (8)	3.5	5	4 (50%)	0.06

The results were also analyzed by groups according to the degree of sexual dysfunction. All the patients (8/8) with severe sexual dysfunction reported difficulty reaching orgasm, with an average score of 3.5 points; after treatment four out of eight patients (50%) showed a reduction in the difficulty of reaching orgasm (mean score of 5); however, this improvement was not statistically significant (P = 0.06).

In the moderate sexual dysfunction group, five out of eight patients (62.5%) experienced difficulty reaching orgasm, with a pre-treatment score of 6.5. After treatment, all patients (5/5) who initially had difficulty reaching orgasm showed improvement, resulting in an average post-treatment score of 8. This improvement was statistically significant (P = 0.039).

Six out of the seven patients with mild sexual dysfunction reported no difficulty in achieving orgasm, with a pre-treatment average score of 8.5 points in this group. The only patient who reported difficulty reaching orgasm (6 points) showed no changes after the intervention (P>0.05).

Frequency of reaching orgasm

Among the 23 patients included in the study, 14 (60.87%) reported infrequent orgasms, with scores of 6 or less. Within this subgroup, four patients (28.57%) reported never achieving orgasms, three patients (21.43%) rarely experienced orgasms, and seven patients (50%) occasionally experienced orgasms.

All patients classified with severe sexual dysfunction (n=8) reported a low frequency of orgasm. Of these, four patients (50%) were unable to achieve orgasm, with an average score of 3.5 in this group. Among patients with moderate sexual dysfunction (n=8), five patients (62.5%) reported decreased orgasmic frequency. Specifically, three patients occasionally experienced orgasms (6 points), one patient rarely achieved orgasm, and another patient reported being unable to achieve orgasm. In contrast, among patients with mild sexual dysfunction (n=7), three patients (42.9%) reported issues with orgasm frequency, of whom two were unable to achieve orgasm, and one rarely experienced it.

Following treatment, a mean increase of 35.44% was observed in the frequency of reaching orgasm among patients with low orgasmic frequency, with scores rising from an average of 4.43 pre-intervention to 6.0 post-intervention. A total of nine out of 14 patients (64.29%) demonstrated improvement in orgasmic symptoms, a change that was statistically significant (P-value 0.0058).

In the group with severe sexual dysfunction, four out of eight patients (50%) showed improvement, with the average score increasing from 3.5 to 5.0. This change was statistically significant (T-statistic: -2.39; P-value: 0.04).

In the group with moderate sexual dysfunction, four out of five patients (80%) with low orgasmic frequency demonstrated improvement, with scores increasing from a mean of 4.8 to 6.4. This improvement was statistically significant (T-statistic: -4.0; P-value: 0.016).

In the group with mild sexual dysfunction, two out of three patients (66.7%) with low orgasmic frequency reported improvement in their scores. One patient did not show improvement, and due to the small sample size, this change did not reach statistical significance (P-value: 0.225) (Table 10).

**Table 10:** Impact of Treatment on Orgasmic Frequency Across Different Degrees of Sexual Dysfunction P-value Calculation: The P-value was determined using a paired t-test to compare baseline and post-treatment scores for the frequency of reaching orgasm. Statistical significance was set at P < 0.05. Baseline Score (Frequency of Reaching Orgasm): Represents the average score reported by patients prior to treatment, measured on a scale from 0 to 10. Post-Treatment Score (Frequency of Reaching Orgasm): Represents the average score reported by patients after the intervention, measured on the same scale. Number of Patients Showing Improvement (%): The proportion of patients who experienced an increase in orgasmic frequency post-treatment, expressed as both the fraction and percentage. Interpretation of P-values: P ≤ 0.05 indicates statistically significant improvement. P > 0.05 indicates that the observed improvement was not statistically significant. Sample Size: Each row corresponds to a specific degree of sexual dysfunction, with the number of patients provided in parentheses.

Degree of Sexual Dysfunction (Number of Patients with Low Orgasmic Frequency)	Baseline Score (Frequency of Reaching Orgasm)	Post-Treatment Score (Frequency of Reaching Orgasm)	Number of Patients Showing Improvement (%)	P-value
All Degrees of Sexual Dysfunction (14 patients)	4.43	6	9/14 patients (64.29%)	0.0058
Grade of Dysfunction (Number of Patients)				
Mild (3 patients)	6.2	6.8	2/3 patients (66%)	>0.05
Moderate (5 patients)	4.8	6.4	4/5 patients (80%)	0.016
Severe (8 patients)	3.5	5	4/8 patients (50%)	0.04

Overall sexual satisfaction

In the category of overall sexual satisfaction, 15 out of 23 patients (65.2%) reported a baseline score of 6 or less, with an average initial score of 5.2 points (Table 11). Among these affected patients, six (40.0% of the affected group; 26.1% of the total cohort) reported sexual dissatisfaction (score of 4), while nine patients (60.0% of the affected group; 39.1% of the total cohort) reported regular satisfaction (score of 6).

**Table 11:** Impact of Treatment on Overall Sexual Satisfaction Across Different Degrees of Dysfunction Baseline Score: The average score for overall sexual satisfaction reported by patients prior to treatment, measured on a scale of 2 to 10. Post-Treatment Score: The average score for overall sexual satisfaction reported by patients following treatment, measured on the same scale. Number of Patients Improved (%): The number and percentage of patients within each subgroup who showed improvement in their overall sexual satisfaction scores after treatment. P-value: The statistical significance of the observed improvement, calculated using a paired t-test. P ≤ 0.05 indicates a statistically significant improvement. P > 0.05 indicates the improvement was not statistically significant. Interpretation: Patients were grouped based on the severity of their sexual dysfunction (mild, moderate, severe) to evaluate treatment efficacy within each subgroup. Percentages were calculated relative to the total number of patients in each subgroup.

Degree of Sexual Dysfunction (Number of Patients with Low Sexual Satisfaction)	Baseline Score	Post-Treatment Score	Number of Patients Improved (%)	P-value
All Degrees of Sexual Dysfunction (15)	5.2	6.6	10/15 (66.7%)	0.0032
Grade of Dysfunction (Number of Patients)				
Mild (1)	8	8	0/1 (0%)	>0.05
Moderate (6)	5.67	8	6/6 (100%)	0.0009
Severe (8)	4.75	5.75	4/8 (50%)	0.17

All patients classified with severe sexual dysfunction (n = 8) reported sexual dissatisfaction, with an average baseline score of 4.75. Among patients with moderate sexual dysfunction (n = 8), six (75.0%) reported dissatisfaction, with an average baseline score of 5.67. In contrast, the group with mild sexual dysfunction (n = 7) had the highest baseline scores, with an average of 8.0 points. Within this group, only one patient (14.3%) reported dissatisfaction, with a baseline score of 6.

In terms of overall sexual satisfaction, the 15 patients with baseline scores indicating low satisfaction (≤6) out of the 23 studied achieved an average score increase from 5.2 to 6.6 points following treatment. This corresponds to an average improvement of 28.2%. A paired t-test confirmed that this change was statistically significant, with a T-statistic of -3.56 and a P-value of 0.0032, highlighting the efficacy of the intervention in addressing sexual dissatisfaction in this subset of patients.

Following treatment, four out of eight patients classified with severe sexual dysfunction demonstrated an improvement in their overall sexual satisfaction scores. The average score in this group increased by 20%, rising from a baseline of 4.75 to 5.75. However, this change did not reach statistical significance (P = 0.170).

All six patients with moderate sexual dysfunction and low baseline overall sexual satisfaction demonstrated an improvement in their scores following treatment. The average score increased from 5.67 to 8.0, reflecting a 41.1% increase in sexual satisfaction. This improvement was statistically significant (P = 0.0009).

Patients with mild sexual dysfunction had an initial score of 8 points (Satisfied) and showed no changes after treatment.

## Discussion

The results of this pilot study suggest that a single session of hybrid CO₂ 10600 nm/1540 nm laser combined with platelet-rich plasma (PRP) demonstrates promising efficacy in improving various domains of female sexual dysfunction, particularly in patients with moderate and severe dysfunction. Statistically significant improvements were observed in overall sexual satisfaction, lubrication, sexual desire, frequency of orgasm, and pain during sexual activity. These findings underscore the potential of this combined approach as an effective therapeutic modality.

Comparison to existing literature

The findings align with prior research on CO₂ laser and PRP therapies for female sexual dysfunction. Previous studies, such as those investigating the MonaLisa Touch system, have demonstrated significant improvements in sexual function, typically requiring three or more treatment sessions to achieve optimal outcomes [6-8, 18]. For example, Athanasiou et al. reported that after three sessions of CO₂ laser therapy, 41% of patients regained normal sexual function, with rates increasing to 69% and 84% following four and five sessions, respectively [18]. Similarly, a systematic review and meta-analysis by Filippini et al. highlighted significant symptom reduction in the genitourinary syndrome of menopause (GSM), including improvements in dyspareunia (mean difference: -5.27, P < 0.001) and FSFI scores (mean difference: 10.8, P < 0.001), with most studies employing three laser sessions [19].

In contrast, our study achieved comparable results after a single session, including a significant 28.2% increase in overall sexual satisfaction (P = 0.0032) and a 41.1% improvement among patients with moderate dysfunction (P = 0.0009). This suggests that combining hybrid CO₂ 10600 nm/1540 nm laser with PRP may enhance tissue regeneration and therapeutic efficacy, potentially reducing the number of sessions required to achieve meaningful clinical outcomes. However, further studies are needed to directly compare this combined approach with standard multi-session CO₂ laser protocols.

These results can also be compared with the work of Dr. Charles Runels [20], who reported improvements in sexual satisfaction (28.5%), lubrication (30.4%), and dyspareunia (22.6%) using the “O-Shot” PRP protocol. Similarly, our study demonstrated significant improvements in these domains, with lubrication increasing by 22.2% (P = 0.0027), and pain during intercourse decreasing by 30.9% (P = 0.0035). Importantly, the addition of hybrid CO₂ 10600 nm/1540 nm laser in our protocol may enhance outcomes by promoting tissue remodeling, as reflected in the 35.4% improvement in orgasmic frequency (P = 0.0058). However, direct comparisons with PRP-only protocols are limited, as no studies combining hybrid laser and PRP have been reported in the literature, highlighting the novelty of this study.

Hormonal status and treatment response

The differential responses between premenopausal and postmenopausal patients are noteworthy. Postmenopausal patients exhibited a significant 30.9% improvement in overall sexual function (P = 0.007), compared to a 7.5% improvement in premenopausal patients (P = 0.04). This disparity may be attributed to the more pronounced baseline dysfunction in postmenopausal women, who had lower average Female Sexual Health Questionnaire scores (34.2 points vs. 46.9 points) [6, 7]. PRP has been shown to enhance vascularization, collagen production, and tissue remodeling through the activation of growth factors such as PDGF, VEGF, and TGF-β, which may partially compensate for estrogen deficits commonly observed in postmenopausal women [19-21]. Dr. Neto’s study on the O-Shot PRP protocol demonstrated significant improvements in lubrication and dyspareunia in postmenopausal patients after two sessions, further supporting the hypothesis that PRP can effectively address symptoms exacerbated by hormonal changes [22]. While encouraging, these findings necessitate further research to validate the observed differences and explore the underlying mechanisms in larger and more diverse cohorts.

Sexual desire

The significant improvements in sexual desire observed in this study further validate the efficacy of the hybrid approach. Among the 15 patients with hypoactive sexual desire, 73.33% showed improvement, with their scores increasing from an average of 4.1 to 6 (P = 0.00048). These results highlight the potential of the combined treatment to address this challenging domain of dysfunction. In the severe dysfunction group, 62.5% of patients demonstrated improvement, with their scores rising from 3.5 to 5.2 (P = 0.041). Notably, in the moderate dysfunction group, all five affected patients improved, and 60% reached scores of 8, categorized as "High sexual desire," with the average score increasing from 4.8 to 7.2 (P = 0.0039). In contrast, the improvements in the mild dysfunction group were minimal, as only one of the two affected patients showed a marginal increase from 4 to 6, which was not statistically significant. These findings align with existing evidence suggesting that greater baseline dysfunction may correlate with more pronounced treatment responses [19, 23, 24].

Lubrication and pain reduction

The significant improvement in lubrication observed in this study, where 54.5% of patients demonstrated enhanced scores (P = 0.0027), aligns with the hypothesized benefits of combining CO₂ laser with PRP. Existing studies support this approach, emphasizing the synergistic effects of these modalities. For instance, Tseluyko and Piskunova reported that combining CO₂ laser with PRP enhanced vaginal hydration and elasticity more effectively than CO₂ laser alone, with improvements in the Vaginal Health Index (VHI) and Female Sexual Function Index (FSFI) persisting for up to 12 months post-treatment after three sessions of CO₂ laser and PRP spaced four weeks apart [23]. Similarly, Behnia-Willison et al. demonstrated that the addition of PRP to CO₂ laser treatments optimized tissue remodeling, promoting collagen synthesis and structural recovery, particularly for patients with vulvovaginal atrophy, also after three sessions [24].

Pain reduction during sexual activity (dyspareunia) also improved significantly in this study, particularly among patients with severe dysfunction. Here, 85.7% reported reduced pain, with a statistically significant improvement in dyspareunia scores (P = 0.0046). This aligns with findings by Gaspar et al., who highlighted that PRP enhanced the regenerative capacity of CO₂ laser treatments, leading to marked reductions in vaginal dryness and dyspareunia after multiple sessions [25].

Notably, the significant results in this study were achieved after a single session of hybrid CO₂ 10600 nm/1540 nm laser combined with PRP, contrasting with the multi-session protocols used in other studies. For example, our study demonstrated a 22.2% improvement in lubrication scores (from 4.91 to 6.0) and a 30.9% reduction in dyspareunia (from 6.2 to 8.13) after one session. In comparison, Tseluyko and Piskunova reported that dyspareunia and dryness resolved completely in their cohort only after three sessions (P < 0.001) [23]. These findings underscore the efficiency of the hybrid laser-PRP approach, potentially reducing patient burden while achieving comparable outcomes to multi-session treatments.

Genital sensitivity and orgasm

Improvements in genital sensitivity and ease of reaching orgasm were observed, though the results varied by the degree of dysfunction. In patients with severe dysfunction, sensitivity improved by 50%, but this change was not statistically significant (P = 0.1). Conversely, patients with moderate dysfunction showed significant improvements in orgasmic frequency, with scores increasing by 35.4% (P = 0.0058). This suggests that the combined treatment effectively enhances sexual responsiveness, particularly in patients with moderate dysfunction, likely due to the synergistic effects of laser-mediated remodeling and PRP-induced regeneration [23-25].

Safety and limitations

The treatment was well-tolerated, with no serious adverse effects reported during the four-week follow-up. This safety profile aligns with existing literature on CO₂ laser and PRP therapies, which report minimal and transient side effects, such as mild irritation or redness [8, 9, 20, 21].

The exploratory nature of this study, the small sample size, and the absence of a control group restrict the generalizability of the findings. These limitations are inherent in pilot studies, which are primarily designed to assess feasibility rather than efficacy or safety and are not typically powered for statistical generalization [26]. Additionally, the heterogeneity of the study population, including differences in hormonal status, baseline dysfunction, and reproductive history, may have influenced treatment responses. The short follow-up period also precludes conclusions about the durability of treatment effects. Future studies should include larger cohorts, longer follow-up periods, and randomized controlled designs to confirm these preliminary findings and establish the long-term efficacy and safety of this combined therapy.

## Conclusions

This pilot study demonstrates that a single session of hybrid CO₂ 10600 nm/1540 nm laser combined with Platelet-Rich Plasma (PRP) offers significant improvements across various domains of female sexual dysfunction, particularly in patients with moderate and severe dysfunction. Statistically significant enhancements were observed in overall sexual satisfaction, lubrication, sexual desire, frequency of orgasm, and pain during sexual activity, highlighting the efficacy of this combined therapeutic approach. This study is the first to explore the combined application of hybrid CO₂ 10600 nm/1540 nm laser and PRP for the treatment of female sexual dysfunction. By integrating the tissue remodeling capabilities of the hybrid laser with the regenerative properties of PRP, this innovative approach has the potential to set a new standard in minimally invasive gynecological therapies. The findings suggest that this hybrid protocol may achieve comparable outcomes to multi-session CO₂ laser treatments while reducing the number of sessions required, thus lowering patient burden and improving treatment accessibility.

Furthermore, the differential responses observed between premenopausal and postmenopausal patients emphasize the potential of PRP in addressing estrogen-deficient conditions, underscoring its regenerative effects. The combined approach demonstrated a favorable safety profile, with no serious adverse effects reported, reinforcing its potential as a minimally invasive treatment option. However, the exploratory nature of this study, the small sample size, and the absence of a control group limit the generalizability of the findings. Future research should focus on randomized controlled trials with larger cohorts and longer follow-up periods to validate these preliminary results and establish the long-term efficacy and durability of this innovative therapeutic modality.

In summary, the hybrid CO₂ laser and PRP approach holds promise as an effective and efficient treatment for female sexual dysfunction, with the potential to significantly advance the field of gynecological regenerative medicine.

## Figures and Tables

**Figure 1:**
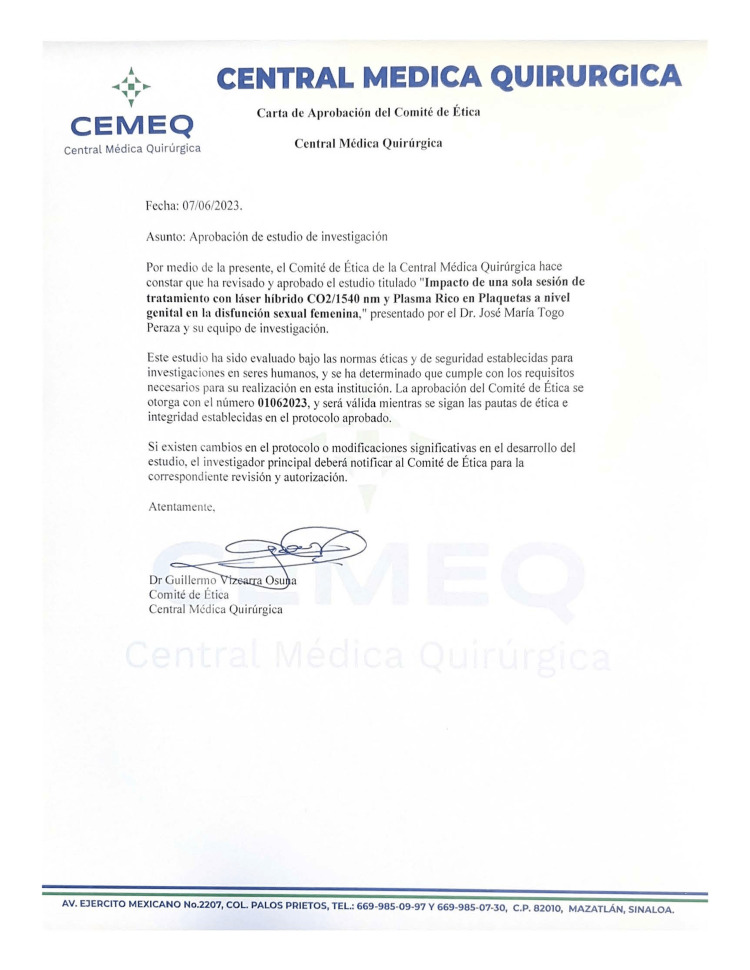
Scanned copy of the IRB approval

## Appendices

FEMALE SEXUAL HEALTH QUESTIONNAIRE

Date:

Patient’s name:

Age:

Session number:

Please select the option that best describes your experience in each aspect before your session. These symptoms assess your last month.

1. How would you rate your desire or interest in having sexual activity?

I never have sexual desire (2 points)

Low (4 points)

Occasionally (6 points)

High (8 points)

Very high (10 points)

2. How would you rate the sensitivity in your genital area during touch or sexual intercourse?

Very insensitive (2 points)

Slightly sensitive (4 points)

Moderately sensitive (6 points)

Sensitive (8 points)

Very sensitive (10 points)

3. How would you rate your level of lubrication during sexual activity?

Very dry (2 points)

Slightly lubricated (4 points)

Lubricated, but not throughout intercourse (6 points)

Lubricated (8 points)

Very lubricated (10 points)

4. How often do you experience orgasms during sexual activity?

I never have orgasms (2 points)

I rarely have orgasms (4 points)

I sometimes have orgasms (6 points)

I frequently have orgasms (8 points)

I always have orgasms (10 points)

5. How would you rate your ease of reaching orgasm?

I don’t have orgasms (2 points)

Very difficult (4 points)

Difficult (6 points)

Easy (8 points)

Very easy (10 points)

6. How would you rate the level of pain you feel during sexual activity?

It’s impossible for me to have sexual intercourse (2 points)

Very painful (4 points)

Tolerable pain (6 points)

Slight pain (8 points)

No pain (10 points)

7. How would you rate your overall level of sexual satisfaction?

Very unsatisfied (2 points)

Unsatisfied (4 points)

Regularly satisfied (6 points)

Satisfied (8 points)

Very satisfied (10 points)

Classification based on the total score for sexual dysfunction:

No sexual dysfunction: 64 - 70 points    

Mild sexual dysfunction: 49 - 63 points

Moderate sexual dysfunction: 35 - 48 points

Severe sexual dysfunction: 14 - 34 points

Glossary of Terms for the Female Sexual Health Questionnaire

Question 1: How would you rate your desire or interest in having sexual activity?

"No sexual desire" (2 points): Complete absence of sexual interest or thoughts.

"Low desire" (4 points): Minimal or infrequent interest in sexual activity; sexual thoughts rarely occur.

"Occasional desire" (6 points): Sporadic interest in sexual activity, occurring under specific circumstances or occasionally initiated by a partner.

"High desire" (8 points): Consistent and regular interest in sexual activity, often initiated spontaneously.

"Very high desire" (10 points): Strong, spontaneous, and frequent interest in sexual activity, including persistent sexual thoughts.

Question 2: How would you describe your sensitivity or sensation during sexual activity?

"No sensitivity" (2 points): Complete lack of genital sensation during sexual activity.

"Low sensitivity" (4 points): Genital sensation is present but weak and not consistently perceived.

"Moderate sensitivity" (6 points): Sensation is noticeable but may not always enhance pleasure.

"High sensitivity" (8 points): Consistent and strong sensation that contributes to sexual pleasure.

"Very high sensitivity" (10 points): Heightened and intense sensation that significantly enhances sexual pleasure.

Question 3: How would you rate the lubrication during sexual activity?

"No lubrication" (2 points): Complete absence of vaginal lubrication.

"Minimal lubrication" (4 points): Vaginal lubrication is present but insufficient or brief.

"Partial lubrication" (6 points): Lubrication is adequate but inconsistent throughout sexual activity.

"Good lubrication" (8 points): Lubrication is sufficient and consistent, with occasional delays.

"Very good lubrication" (10 points): Lubrication is immediate, sustained, and fully adequate throughout sexual activity.

Question 4: How would you rate the pain experienced during sexual activity?

"Severe pain" (2 points): Intense and distressing pain that prevents sexual activity.

"Moderate pain" (4 points): Noticeable discomfort that may limit sexual activity.

"Tolerable pain" (6 points): Pain is present but manageable, allowing continued sexual activity.

"Mild pain" (8 points): Slight discomfort that does not interfere significantly with sexual activity.

"No pain" (10 points): Sexual activity is completely pain-free.

Question 5: How often do you reach orgasm during sexual activity?

"Never" (2 points): Complete inability to achieve orgasm.

"Rarely" (4 points): Achieves orgasm occasionally, less than 25% of the time during sexual activity.

"Occasionally" (6 points): Achieves orgasm around 50% of the time during sexual activity.

"Frequently" (8 points): Achieves orgasm consistently, around 75% of the time during sexual activity.

"Always" (10 points): Achieves orgasm every time during sexual activity.

Question 6: How easy is it for you to reach orgasm during sexual activity?

"Very difficult" (2 points): Achieving orgasm requires significant effort and prolonged stimulation.

"Difficult" (4 points): Achieving orgasm is challenging and requires focused effort.

"Moderate effort" (6 points): Achieving orgasm requires some effort but is generally possible.

"Easy" (8 points): Achieving orgasm is natural and occurs with minimal effort.

"Very easy" (10 points): Achieving orgasm is effortless and occurs spontaneously during sexual activity.

Question 7: How would you rate your overall satisfaction with your sexual life?

"Very dissatisfied" (2 points): Complete dissatisfaction with sexual experiences.

"Dissatisfied" (4 points): Frequent dissatisfaction with sexual experiences.

"Occasionally satisfied" (6 points): Mixed satisfaction with some positive and negative experiences.

"Satisfied" (8 points): Consistent satisfaction with sexual experiences.

"Very satisfied" (10 points): Complete satisfaction and fulfillment with sexual experiences.
